# Vitamin A cycle byproducts explain retinal damage and molecular changes thought to initiate retinal degeneration

**DOI:** 10.1242/bio.058600

**Published:** 2021-11-29

**Authors:** Dan Zhang, Doina M. Mihai, Ilyas Washington

**Affiliations:** 1 Columbia University Medical Center, Ophthalmology, New York, NY 10032, USA; 2 biOOrg3.14, Buffalo, WY 82834, USA

**Keywords:** Vitamin A, Retina, A2E, Age-related macular degeneration, Stargardt disease

## Abstract

In the most prevalent retinal diseases, including Stargardt disease and age-related macular degeneration (AMD), byproducts of vitamin A form in the retina abnormally during the vitamin A cycle. Despite evidence of their toxicity, whether these vitamin A cycle byproducts contribute to retinal disease, are symptoms, beneficial, or benign has been debated. We delivered a representative vitamin A byproduct, A2E, to the rat's retina and monitored electrophysiological, histological, proteomic, and transcriptomic changes. We show that the vitamin A cycle byproduct is sufficient alone to damage the RPE, photoreceptor inner and outer segments, and the outer plexiform layer, cause the formation of sub-retinal debris, alter transcription and protein synthesis, and diminish retinal function. The presented data are consistent with the theory that the formation of vitamin A byproducts during the vitamin A cycle is neither benign nor beneficial but may be sufficient alone to cause the most prevalent forms of retinal disease. Retarding the formation of vitamin A byproducts could potentially address the root cause of several retinal diseases to eliminate the threat of irreversible blindness for millions of people.

## Abstract (precis)

**Summary:** During the vitamin A cycle, byproducts of vitamin A form in the eye. Using a rat model, we show that the byproducts alone can explain several retinal derangements observed in the prodromal phase of human retinal disease. Retarding the formation of these byproducts may address the root cause of the most prevalent retinal diseases.

## INTRODUCTION

Atrophic lesions involving the photoreceptors, retinal pigment epithelium (RPE), choriocapillaris and loss of vision characterize end-stage Stargardt disease and age-related macular degeneration (AMD). According to histopathological analysis, atrophic lesions began with atrophic changes to the RPE, which spread to the choriocapillaris and Bruch membrane, and overlying neuroretina (Bertolotto et al., 2014; Bird, 2020; Birnbach et al., 1994; Eagle et al., 1980; Ferris et al., 1984; Hogan, 1972; Sarks et al., 1988; Sarks, 1976). In Stargardt disease and AMD, pre-symptomatic RPE changes can also be observed via optical coherence tomography (Curcio et al., 2017), along with atrophic changes to the ellipsoid zone (EZ, the photoreceptor inner segment/outer segment junction) (Cai et al., 2018; Toprak et al., 2017), and expansion (Lamin et al., 2019) and thinning (Whitmore et al., 2020) of the outer plexiform layer (OPL).

The above morphological changes are concurrent with various molecular derangements. The retina also exhibits signatures suggestive of altered states of immunity (Bora et al., 2014), autophagy (Kaarniranta et al., 2013), mitochondrial function (Kenney et al., 2013), and oxidative metabolism (Ugurlu et al., 2013), to name a few. A challenge in elucidating the pathophysiology of retinal disease is deconvoluting between events that drive the degenerative processes and events that are secondary symptoms.

Retinal poisons, such as ornithine and sodium iodate, can also cause several of the above morphological and molecular changes and result in atrophic lesions similar to those seen in late-stage Stargardt disease and AMD (Machalinska et al., 2010; Maeda et al., 1998). This work proposes that the atrophic lesions observed in retinal diseases such as Stargardt disease and AMD can be explained by a poisoning from the chronic formation of vitamin A byproducts during the vitamin A cycle.

During the usual process to enable vision, the vitamin A cycle, an unknown portion of vitamin A, is its retinaldehyde form, reacts with phosphatidylethanolamine or lysine (Eldred and Lasky, 1993; Fishkin et al., 2003, 2005; Katz et al., 1996; Sakai et al., 1996) and another molecule of retinaldehyde to generate dimers of retinaldehyde. These dimers of retinaldehyde can be oxidized, transformed into oxidative catabolites (Ben-Shabat et al., 2002a; Ueda et al., 2016; Wang et al., 2006; Washington et al., 2006, 2005; Wu et al., 2010; Yoon et al., 2012), and/or react with additional retinaldehyde molecules to generate higher-order oligomers (Murdaugh et al., 2011, 2010b). These dimers and oligomers of retinaldehyde can be found in the RPE (Eldred and Katz, 1988; Eldred and Lasky, 1993; Mihai and Washington, 2014; Sakai et al., 1996) and Bruch's membrane (Murdaugh et al., 2010a) of the eye. An obstacle in understanding diseases of the retina has been elucidating the significance of the formation of these vitamin A cycle byproducts. As a model byproduct, A2E is often used because it is characterized, can be synthesized in a lab, and extracted and quantified from eyes (Penn et al., 2014). However, there are presumably hundreds of waste byproducts and oligomer aggregates derived from vitamin A. Over the past three decades, several mechanisms have been proposed by which byproducts of the vitamin A cycle, particularly A2E, might contribute to retinal pathology (Anderson et al., 2013; Avalle et al., 2004; Ben-Shabat et al., 2002a,b; Bermann et al., 2001; De and Sakmar, 2002; Dontsov et al., 2009; Finnemann et al., 2002; Holz et al., 1999; Iriyama et al., 2009, 2008; Kanofsky et al., 2003; Lakkaraju et al., 2007; Lukiw et al., 2006; Ma et al., 2013; Moiseyev et al., 2010; Murdaugh et al., 2009, 2010a; Nowak, 2013; Radu et al., 2004; Roberts et al., 2002; Sokolov et al., 2007; Sparrow, 2010; Sparrow and Cai, 2001; Sparrow et al., 2000, 2003a, 1999, 2006, 2002, 2003b; Thao et al., 2013; van der Burght et al., 2013; Vives-Bauza et al., 2008; Wang et al., 2006; Washington et al., 2005; Wielgus et al., 2010; Wu et al., 2010; Yoon et al., 2011, 2012; Zhou et al., 2006). Nevertheless, the contribution of the vitamin A byproducts to retinal health is debated, and their formation has even been argued to be beneficial (Roberts et al., 2002).

We have observed that exposing cultured RPE cells to vitamin A byproducts triggered similar pathology to that observed in the aging *in vivo* RPE (Mihai and Washington, 2014). Further, we observed that exogenous delivery of the A2E byproduct to the *in vivo* rabbit eye triggered retinal degeneration in a similar sequence observed in humans (Penn et al., 2014). Iriyama et al. injected A2E into the subretinal space of the *in vivo* mouse eye and concluded that the vitamin A dimer was pro-angiogenic (Iriyama et al., 2009). Accordingly, we sought to determine the extent to which the A2E vitamin A byproduct, delivered exogenously to the living eye, triggered the functional, morphological, and molecular derangements observed in human retinal disease.

By delivering A2E into the rat eye, via intravitreal injection, we show that the vitamin A byproduct is likely detrimental. Data reveals that the formation the vitamin A cycle byproducts during the vitamin A cycle is sufficient alone to explain the functional, morphological, and molecular phenotypes observed during the most prevalent forms of retinal degeneration. The data suggests that retarding the formation of the vitamin A byproducts could result in a disease-modifying intervention.

## RESULTS

### Intravitreally injected A2E reaches the RPE and the neural retina

**Fig. 1.**
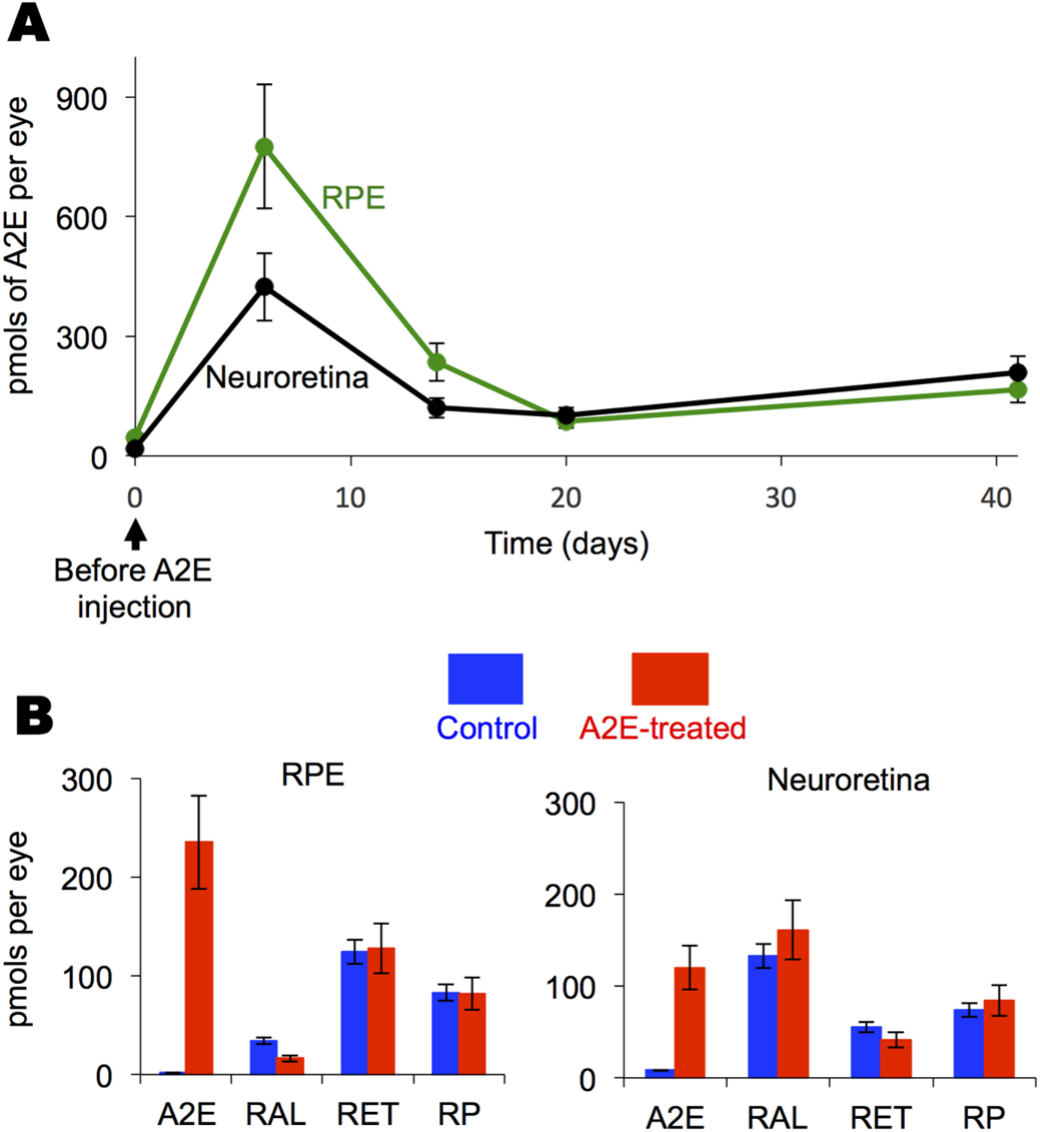
**Intravitreally injected A2E reaches the RPE and the neural retina.** (A) Average amounts of A2E with standard deviations in the RPE and neural retina. *n*=2 to 4 eyecups at each point. A2E (10 µg) was delivered intravitreally at day zero. At the shown times RPE and neural retina were collected and A2E quantified by HPLC. (B) Expanded data for the 2-week time point shown in panel A showing average amounts of A2E and vitamin A congeners, with standard deviations, in the RPE and neuroretina of the A2E-treated and sham treated cohorts, *n*=3 eyes. RAL, Retinaldehyde; RET, retinol; RP, retinal palmitate.

Because intravitreally delivered materials tend to accumulate in the RPE (Katz et al., 1999; Tabatabay et al., 1987; Yasukawa et al., 2007), we injected A2E into rats’ vitreous chamber. Before administering A2E, the rat RPE and neuroretina contained about 50 and 20 pmols of A2E per eye, respectively, in accordance with literature reports (Reinboth et al., 1997). Six days after the intravitreal injection of A2E, the concentrations of A2E increased by 16 times in the RPE and 24 times in the neuroretina (Fig. 1A). The concentrations of A2E in both the RPE and neuroretina decreased and leveled off when measured 20 days after injection (Fig. 1A). The relative concentrations of the major ocular vitamin A congeners remained unchanged in the A2E-treated eyes, relative to control eyes injected with the vehicle only (Fig. 1B), when measured 2 weeks after injection, indicating normal retinal homeostasis in regards to vitamin A. From about 2 weeks after injection onward, the ratio of A2E relative to vitamin A (the sum of retinol, retinaldehyde, retinyl esters) in the A2E-treated eyes was similar to that found in humans after the seventh decade of life (Zhang et al., 2021).

### The vitamin A cycle byproduct, A2E, delivered intravitreally induces slow functional declines

**Fig. 2.**
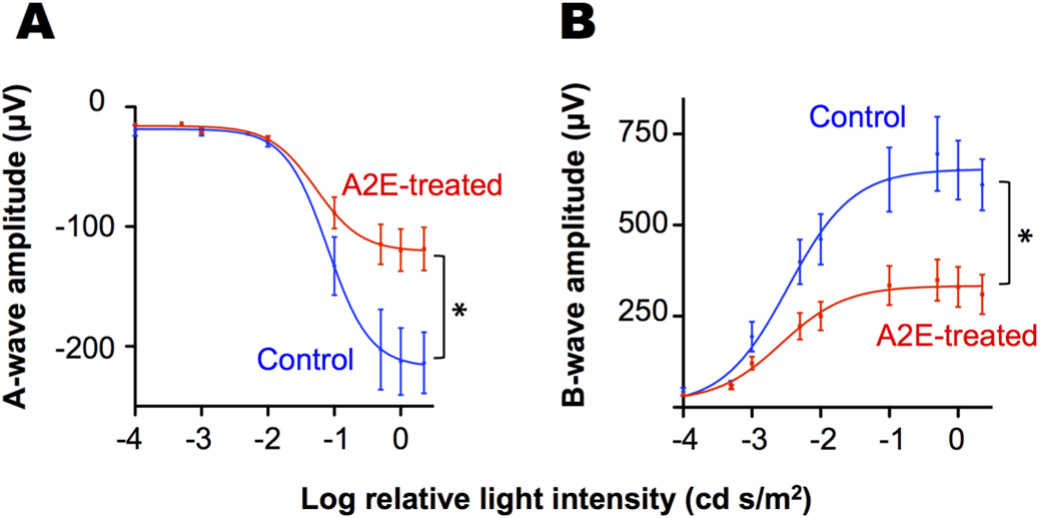
**The vitamin A cycle byproduct, A2E, delivered intravitreally induces functional declines.** Average (A) a-wave and (B) b-wave ERG amplitudes in response to flashes of light of increasing intensity for rats treated with A2E 2 months prior (A2E-treated, *n*=18 animals) along with ERG amplitudes of sham-injected, age-matched control rats (Controls, *n*=6 animals). Averages and 95% confidence intervals are shown. * *P*-value <0.05 as determined by a *F*-test.

Two months after A2E treatment, we evaluated retinal function via electroretinography. The ERG measures the electrical response of the eye elicited by flashes of light. ERG parameters, the amplitudes of the a- and b-waves, are used as indicators of retinal function. The average maximum a-wave was reduced by 44% in the A2E-treated animals compared to controls (−217±17 μV in control versus −121±8 μV in A2E-treated, *P*=<0.001) and the intensity of light needed to elicit half of the maximum a-wave was 33% greater in the A2E-treated animals (0.079 cd s/m^2^ in control versus 0.053 cd s/m^2^ in A2E-treated, *P*=<0.05) (Fig. 2A). Likewise, maximum b-wave of the A2E-treated animals was depressed by approximately 50% of that of control animals (653±38 μV in control versus 333±23 μV in A2E-treated, *P*=<0.001) and the intensity of light needed to elicit half of the maximum b-wave was 18% greater in the A2E-treated animals (0.0033 cd s/m^2^ in control versus 0.0027 cd s/m^2^ in A2E-treated, *P*=<0.05) (Fig. 2B). In contrast, depressed ERG a- and b-waves were not observed 7 days after injection, indicating that A2E causes a slow decline in retinal function instead of immediate retinal damage as observed for RPE poisons, such as sodium iodate and ornithine.

### The vitamin A cycle byproduct, A2E, delivered intravitreally induces slow morphological changes

**Fig. 3.**
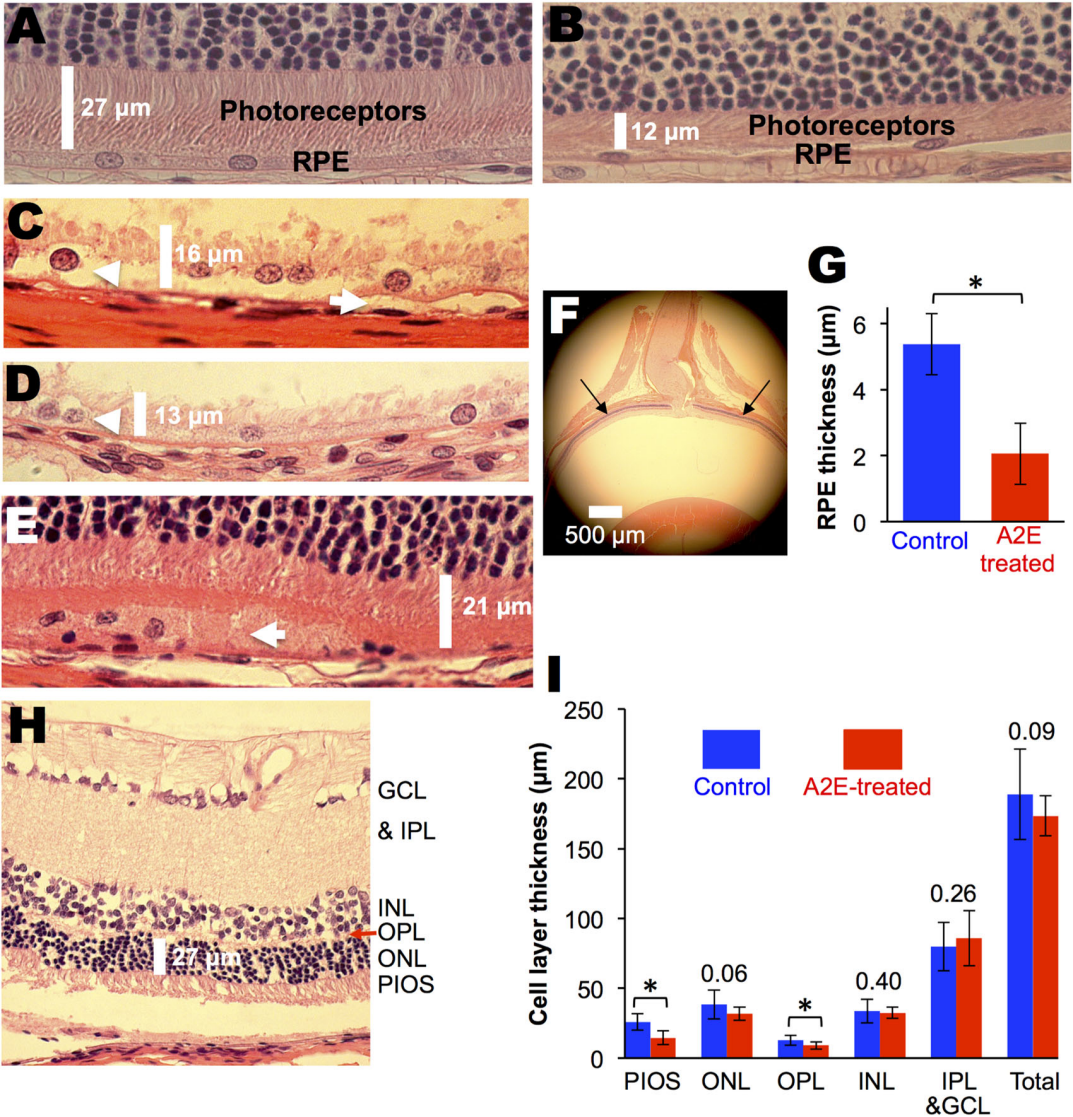
**The vitamin A cycle byproduct, A2E, delivered intravitreally induces morphological changes, albino animals.** (A) Control albino Wistar rat showing normal histology. (B) A2E-treated animal showing an atrophied RPE and photoreceptor layer. (C-E) A2E-treated animals showing accumulation of debris under, around and in the RPE layer. Arrows point to A2E induced changes. (F) Location of measured retinal thickness, 1 mm from both sides of the optic nerve head. (G) Average RPE thickness in A2E-treated (*n*=8 eyes) and control (*n*=4 eyes) eyes. (H) Representative retinal section showing retinal layers. PIOS, photoreceptor inner and outer segments; ONL, outer nuclear layer; OPL, outer plexiform layer; INL, inner nuclear layer; IPL, inner plexiform layer; GCL, ganglion cell layer. (I) Average retinal cell layer thickness in A2E-treated (*n*=4 eyes) and control (*n*=4 eyes) eyes. For panels I and G, averages and standard deviations are shown. Numbers above bars are *P*-values comparing controls with A2E-treated. * *P*-value <0.05 as determined by a *t*-test.

Morphological changes induced by A2E, 2 months after an injection, were most prevalent in the RPE (Fig. 3). The average thickness of the RPE in A2E-treated animals, measured 1 mm from both sides of the optic nerve head, is shown in Fig. 3G and was 60% thinner compared to control animals (5±1 μm in controls versus 2±1 μm in A2E-treated, *P*=<0.01). Cellular debris and swelling were detected in and around the RPE in the A2E-treated animals (Fig. 2A), which were absent in control retinas. Signs of pathology were also observed in the photoreceptor inner and outer segments (PIOS) and outer plexiform layer (OPL). The average thickness of the PIOS in the A2E-treated animals was 42% thinner compared to control animals (26±6 μm in controls versus 15±5 μm in A2E-treated, *P*=<0.001), and the average thickness of the OPL, was 31% thinner compared to control animals (13±4 μm in controls versus 9±3 μm in A2E-treated, *P*=<0.05). Outer nuclear layer (ONL) nuclei were found in the OPL and PIOS in the A2E treated animals, relative to controls; this difference was not quantified (Gartner and Henkind, 1981). The thicknesses of the remaining retinal layers were statistically the same in the control and A2E-treated animals. As with ERG amplitudes, morphological changes were not observed 5 days after injection.

**Fig. 4.**
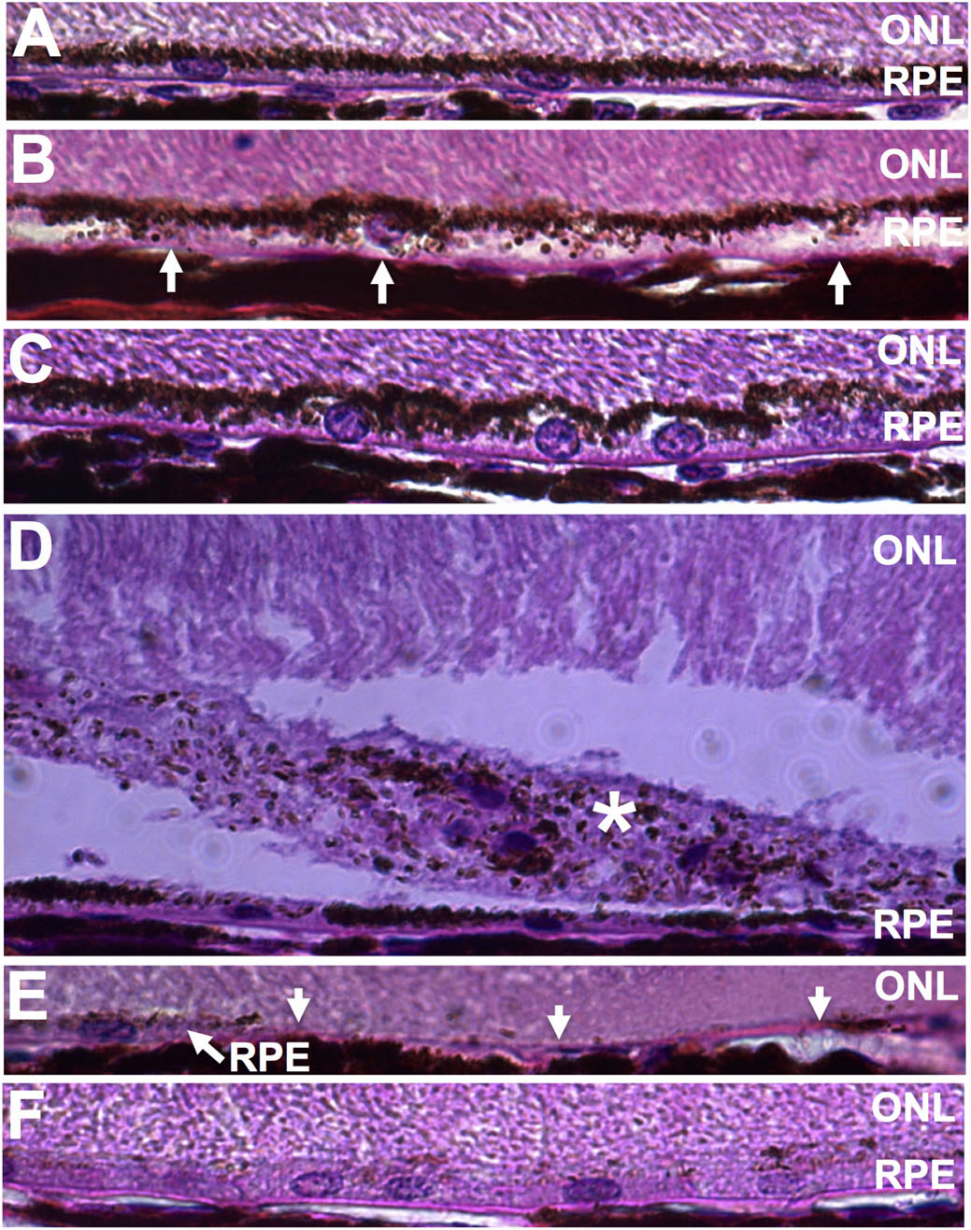
**The vitamin A cycle byproduct, A2E, delivered intravitreally induces morphological changes, pigmented animals.** (A) Control Long-Evans rat showing normal RPE histology. (B-C) RPE swelling. (D) Star indicates subretinal debris. (E) Arrows indicate missing RPE. (F) Hypopigmentation of the RPE.

Because vitamin A byproducts are physically associated with melanin granules, we also compared histological changes in pigmented rats treated with intravitreal A2E with age-matched, vehicle-treated eyes (Fig. 4A). Similar morphological changes, such as RPE swelling (Fig. 4B), subretinal debris (Fig. 4C), and RPE degeneration (Fig. 4E), were seen in the pigmented rats. In addition areas of hypo-pigmentation were observed in the pigmented rats (Fig. 4F). The neuroretina was not significantly altered. All five A2E-treated eyes displayed some RPE pathology as described above, while none of the four control eyes displayed RPE pathology. A2E was significantly correlated RPE damage by histology [OR (95%CI)=0.01 (0.0002 to 0.6); *P*-value=0.0079].

### The vitamin A cycle byproduct, A2E, delivered intravitreally induces molecular changes observed in human retinal disease

**Fig. 5.**
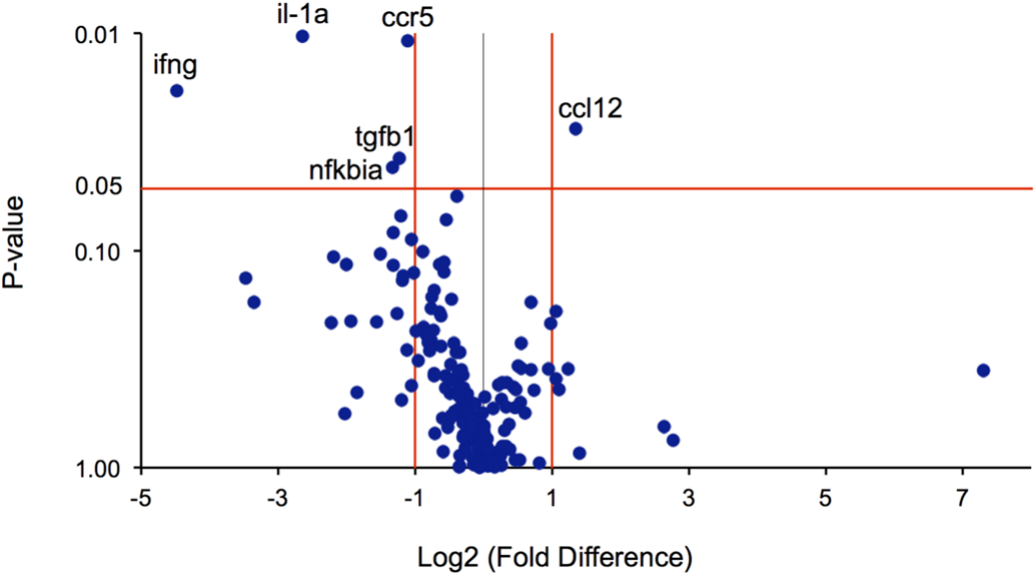
**The vitamin A cycle byproduct, A2E, delivered intravitreally alters gene transcription.** Volcano plot depicting average fold change with statistical significance (*P*-value) in mRNA transcripts corresponding to 167 genes involved in inflammation and autophagy in eyes (*n*=6 eyes) treated with A2E 8 weeks prior, compared to age-matched control eyes (*n*=10 eyes). Genes names are shown for transcripts with a statistically significant change (*P*<0.05 red horizontal line), greater than twofold (red vertical lines). Il-1a, interleukin 1 alpha; Ccr5, chemokine (C-C motif) receptor 5, involved in adaptive immunity; Nfkbia, nuclear factor of kappa light polypeptide gene enhancer in B-cells inhibitor, alpha, which is involved in innate immunity; Ccl12: chemokine (C-C motif) ligand 12, which is involved in humoral immunity; Ifng, interferon-γ, which also plays a role in immunity. Tgfb1, cytokine, regulators of autophagy and apoptosis, transforming growth factor, beta 1, also play roles in immunity.

To determine if the above electrophysiological and morphological changes correlated with molecular dysregulation, we used quantitative reverse transcription-polymerase chain reaction (qRT-PCR) to survey the expression of 167 genes involved in inflammation and autophagy. Out of the 167 genes surveyed, 27 transcripts were downregulated greater than twofold, and 9 transcripts were upregulated greater than twofold in the A2E-treated retinas relative to the controls, suggesting molecular dysregulation (Fig. 5). Of the 27 down-regulated transcripts, five were downregulated with statistical significance: interleukin 1 alpha (IL-1a) and nuclear factor of kappa light polypeptide gene enhancer in B-cells inhibitor, alpha (Nfkbi-a), which are involved in innate immunity; the chemokine (C-C motif) receptor 5 (Ccr5), involved in adaptive immunity; interferon-gamma (Ifng, which is involved in both innate and adaptive immunity; and Transforming growth factor, beta 1 (Tgfb-1). One gene was significantly upregulated: chemokine (C-C motif) ligand 12 (CCL12).

**Fig. 6.**
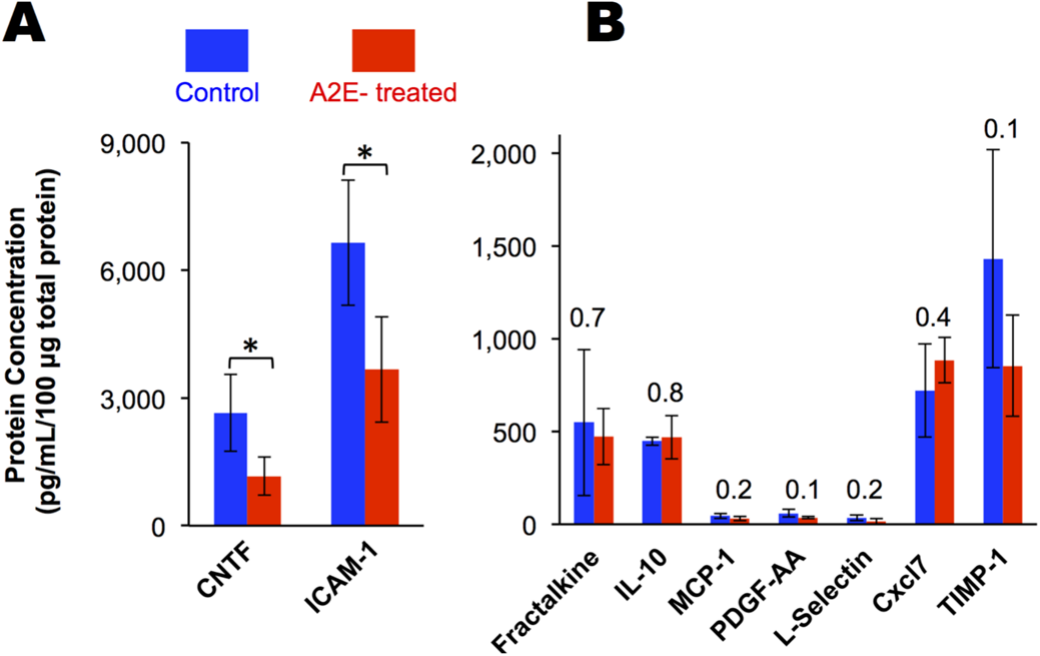
**The vitamin A byproduct, A2E, delivered intravitreally alters the inflammatory status of the retina.** Cytokines in A2E-treated eyes (*n*=4 eyes) relative to control eyes (*n*=4 eyes) quantified by ELISA in homogenized retina. CNTF, ciliary neurotrophic factor; ICAM-1, intercellular adhesion molecule 1; IL-10, interleukin 10; MCP-1, monocyte chemotactic protein; PDGF-AA, platelet-derived growth factor alpha polypeptide; Cxcl7, Chemokine (C-X-C motif) ligand; TIMP-1, tissue inhibitor of metalloproteinases 1. Averages and standard deviations are shown. Numbers above bars are *P*-values comparing controls and treated. * *P*-value <0.05 as determined by a *t*-test.

Next, we quantified levels of nine cytokines via enzyme-linked immunosorbent assay (ELISA) (Fig. 6). A2E-treated animals had 56% less ciliary neurotrophic factor (CNTF), (2649±904 pg/ml in controls versus 1164±446 pg/ml in A2E-treated, *P*=<0.001) and 45% less intercellular adhesion molecule 1 (ICAM-1) (6644±1465 pg/ml in controls versus 3668±1237 pg/ml in A2E-treated, *P*=<0.001) compared to control animals. The concentrations of the other seven cytokines surveyed were statistically the same in both the control and A2E-treated animals (Fig. 5B).

**Fig. 7.**
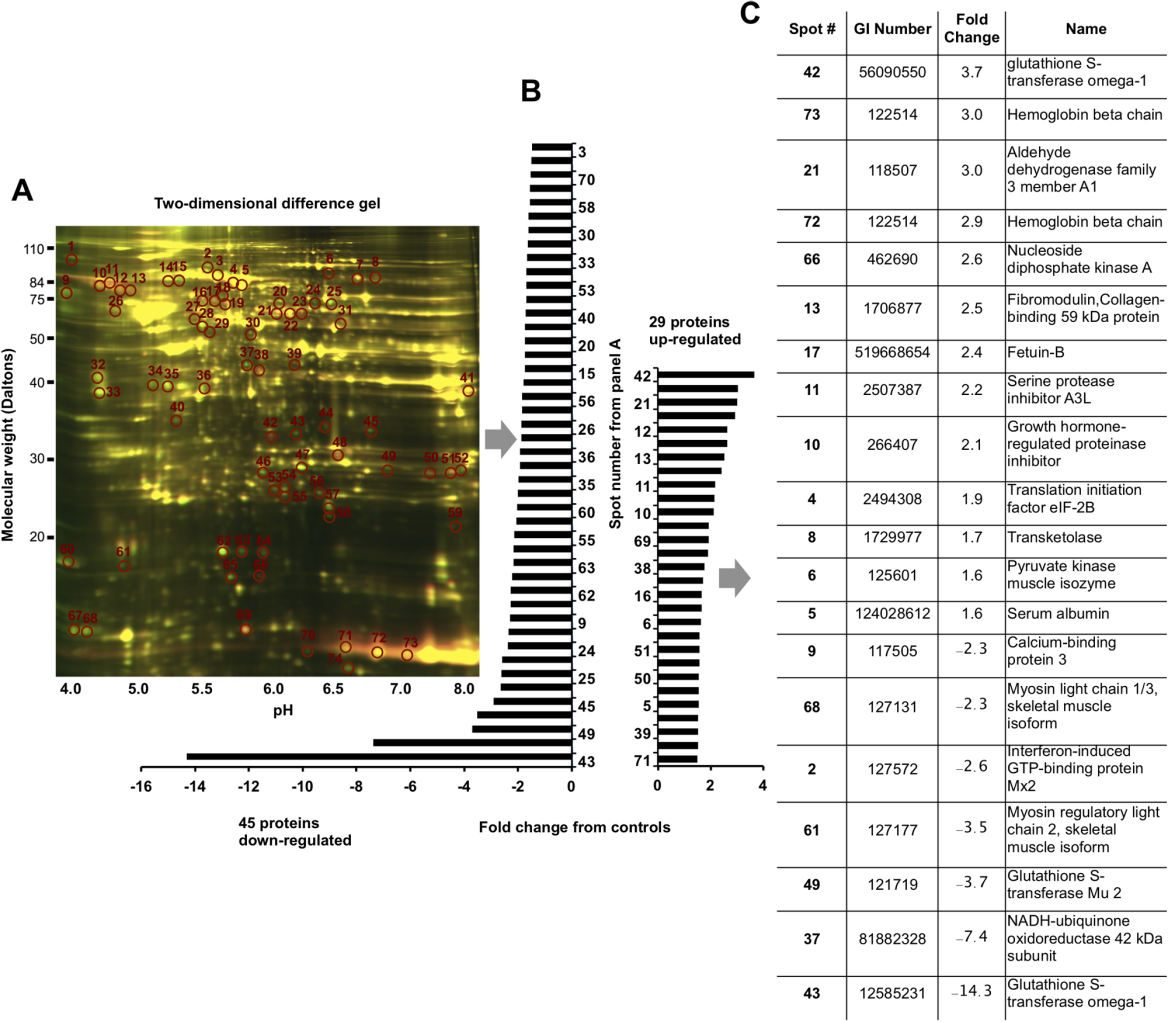
**The vitamin A cycle byproduct, A2E, delivered intravitreally dysregulates protein synthesis.** (A) Difference gel electrophoresis of proteins isolated from the retina and eyecup of control (*n*=4 eyes pooled) and A2E-treated (*n*=4 eyes pooled) rats. Differentially expressed proteins are circled. Proteins down- and upregulated are summarized in panel. (B) Of the 74 differentially synthesized proteins, 20 were identified by mass spectrometry and listed in panel C.

We further surveyed total protein synthesis in the A2E-treated and control eyes using two-dimensional (2D) difference gel electrophoresis. We were able to detect over 2000 unique proteins in the homogenized eyecups. Of these proteins, 45 were downregulated, and 29 were upregulated in the A2E-treated eyes compared to the control eyes, in accord with an overall downregulation in transcription and the downregulation of CNTF and ICAM-1. We used mass spectrometry to identify 20 of the 74 differentially regulated proteins with greater than 99% confidence (Fig. 7C). When these 20 proteins were classified according to related gene ontology (GO), 9 of the 20 proteins were involved in metabolic processes (GO:0008152), 4 of the 20 proteins were involved in cellular processes (GO:0009987), biological regulation (GO:0065007), or multicellular organismal process (GO:0032501). Pathway analysis revealed that 6 canonical pathways might have been influenced by differential expression of 4 of the 20 identified proteins. These pathways with their pathway accession numbers and corresponding proteins were as follows: the pyrimidine ribonucleotides biosynthesis pathway (P02740) protein NDKA; the pyrimidine deoxyribonucleotide biosynthesis pathway (P02739) protein NDKA; the purine biosynthesis pathway (P02738) protein NDKA; the pentose phosphate pathway (P02762) protein TKT; the 5-hydroxytryptamine degradation pathway (P11883) protein AL3A1; and the pyruvate metabolism pathway (P02772) protein KPYM.

## DISCUSSION

No model can reproduce every aspect of human retinal degeneration: as retinal degenerations are heterogeneous, neither does any one human. To model the formation of the vitamin A byproducts, we delivered the vitamin A byproduct A2E via intravitreal injection into the rat. With the use of the whole eye, biological cascades involved in inflammation, apoptosis, and necrosis, which occur during human retinal degeneration, can potentially be modeled.

*In vivo*, the vitamin A byproducts form in the RPE and the outer nuclear layer, where retinaldehyde enters or exits opsin-binding sites. In contrast, intravitreal delivery exposes the entire neural retina to A2E. Despite the whole retina being exposed, marked morphological changes were mainly observed in the RPE, with thinning of the PIOS and OPL. The histological changes to the RPE occurred over months and were not acute, in accord with the slow histological initiation and progression of Stargardt disease and AMD. The similar sequence of pathology and slow timing of the pathology suggest some utility for this intravitreal injection model.

The cause of the PIOS and OPL thinning is not apparent. The thinning may be in response to the A2E-damaged RPE or a direct A2E-induced damage to the neuroretina. As degeneration of the neural retina does not necessarily lead to deterioration of the PRE, as exemplified by the RCS rat (Ryals et al., 2017), the observed RPE damage is unlikely to be a result of A2E-induced damaged to the neural retina. We did not investigate the choriocapillaris. Early damage to choriocapillaris has been argued to drive retinal degeneration (Biesemeier et al., 2014). The choriocapillaris and RPE are mutually dependent: damage to one leads to damage in the other. Previous work has shown that vitamin A cycle byproducts, delivered to the retina, damage the choriocapillaris (Iriyama et al., 2009; Penn et al., 2015). At this stage, it is not clear whether the delivered A2E preferentially damaged the choriocapillaris or RPE, which resulted in subsequent damage of the other, or if both structures were equally damaged primarily.

We interrogated the retina for molecular signatures 2 months after delivery of A2E when ERG amplitudes were reduced by approximately 50%. Several proteins that were dysregulated in this model are also dysregulated during human retinal degeneration. For example, CNTF, a survival factor for neurons and oligodendrocytes, was downregulated here and downregulated in AMD (Harris et al., 2013). Glutathione S-transferase (GST) showed the largest protein expression changes, both up- and downregulated, upon treatment with A2E, spots 42, 43, and 49 in Fig. 7A. A single protein can resolve into multiple spots, reflecting functionally distinct subpopulations due to proteolytic processing, isoforms, or changes in charge resulting from posttranslational modifications (phosphorylation, deamidation, acetylation, glycation, and glutathionylation), indication that treatment induced different subpopulations of the protein. GST expression is regulated by retinoid X receptor (RXRA) (Dai et al., 2005), which is in turn modulated by A2E (Iriyama et al., 2008). The downregulation of GST and the upregulation of pyruvate kinase observed here also occur during AMD's progressive stages (Nordgaard et al., 2006). The second-most upregulated protein was hemoglobin, which is synthesized by the RPE (Tezel et al., 2009). Deposition of hemoglobin within the matrix of Bruch's membrane is significantly associated with retinal senescence (Beattie et al., 2010). Furthermore, hemoglobin and serum albumin ­– upregulated here – are both found in human drusen, a hallmark of AMD eyes (Crabb et al., 2002). As hemoglobin is used for retinal oxygenation, an upregulation of hemoglobin is consistent with a hypoxic environment. Along these lines, fibromodulin, a stimulator of angiogenesis, was also upregulated in A2E-treated eyes and is upregulated in the eyes of people with AMD (Adini et al., 2014). The upregulation of fibromodulin and hemoglobin is consistent with A2E promoting an angiogenic environment responsible for wet-AMD and consistent with previous work suggesting that exogenous A2E can lead to vesicle-like structures in the rabbit retina. Lastly, quantification of mRNA (Fig. 4) and ICAM-1, a cell surface protein that is upregulated upon cytokine stimulation, suggested that A2E triggered a general downregulation of transcripts involved in immunity. Dysregulation in both the innate and adaptive immune pathways is thought to be involved in the etiology of AMD (Bora et al., 2014; Camelo, 2014). Although excessive transcription of immunity-related proteins has been considered harmful, a reduction in transcription may represent an immunodeficiency. Suppression of specific immune pathways could trigger a subsequent upregulation of alternative immune pathways (Ertel et al., 1995). The observation that the majority (9 out of 20) of the proteome changes involved metabolic pathways indicates that the highly metabolic photoreceptors may be affected.

Interrogation of molecular signatures at earlier or later time points would presumably have presented different molecular states. Further, different species, including humans, may respond differently to A2E-induced toxicity. In Stargardt disease, the vitamin A cycle byproducts form abnormally in the young retina. In AMD, the byproducts form, to a similar degree, in the aged retina. The proposed work would conclude that the difference in the two diseases is explained by the age of the eye in which the vitamin A cycle byproducts form. The major relevance of the presented transcriptional and proteomic dysregulation, at this time, is that the vitamin byproduct, A2E, was sufficient alone to cause them.

Data are consistent with the theory that the formation of the vitamin A byproducts is neither benign nor beneficial. This work demonstrates that vitamin A byproducts are capable of initiating changes observed in the prodromal phase of Stargardt and AMD. It presumes, like the aforementioned retinal poisons, that these changes would progress to atrophic lesions, given adequate time, a greater concentration of the vitamin byproducts, or a different biological system. Accordingly, we propose that the morphological and molecular derangements that characterize retinal degenerations, marked by the formation of vitamin byproducts, can be explained by a chronic poisoning from the byproducts. If so, therapeutic interventions that target downstream reactions to the byproducts, without addressing their initial formation, will be limited in therapeutic value. The continued poising will eventually overwhelm any therapeutic block or divert the system to alternative pathways towards degeneration. A therapeutic that retards the formation of the vitamin A cycle byproducts may be necessary to significantly alter the course of these diseases.

## MATERIALS AND METHODS

### Animals

All experiments were conducted in accordance with the current *Guide for the Care and Use of Laboratory Animals*, published by the US National Academies Press. Columbia University's Institutional Animal Care and Use Committee (IACUC) approved animal protocols. We used 3-month-old Fischer crossbred Wistar rats for the albino rats and Long Evans rats (Charles River) for the pigmented animals. Roughly an equal number of male and female animals were used. Animals were allocated to experimental groups at random. No animals were excluded from analysis.

### A2E administration

A2E was prepared as previously described (Penn et al., 2014). A2E was dissolved in dimethyl sulfoxide (DMSO). This stock solution was added to a solution of phosphate-buffered saline (PBS) that was probe sonicated during the addition to give a solution of A2E (1 mg A2E/ml PBS) containing 1% DMSO. Ten microliters of the solutions were injected intravitreally into each eye. For controls, a solution of PBS with 1% DMSO in PBS was injected.

### A2E quantification

Eyes were enucleated and stored at −20°C in the dark before analysis. Either one or two eyes were placed into 1.5 ml of homogenization tube and minced 5-8 times with scissors. Added to the tube were: 500 μl of brine, 10 μl of 37% paraformaldehyde, 400 μl of ethyl acetate (containing 1 mg butylated hydroxytoluene per ml of ethyl acetate) and 0.5 g of stainless-steel beads, 0.9-2 mm in diameter. The mixture was homogenized with a bead mill homogenizer (BBY24M Bullet Blender^®^ Storm, Next Advance, Inc., Averill Park, NY, USA; setting speed of 8 for 15 min) and centrifuged for 5 min at 14,000 rpm. We removed 200 μl of the butanol phase and added it to a 300 μl capacity autosampler vial (Microsolv Technology Corp., Eatontown, NJ, USA). Samples were held at room temperature in an autosampler until they were injected in the ultra-performance liquid chromatography (UPLC) system.

For UPLC analysis, we injected 30 μl of the above eye extracts into a Flexar FX-15 system with a PDA Detector (PerkinElmer, Waltham, MA, USA). The system contained a 2.1 mm×50 mm, 2.1 μm particle size, C18 column (Brownlee SSP, PerkinElmer) with a 2.1 mm×5 mm guard column with the same packing material (Waters Corp., Milford, MA, USA). The column oven was set to 40°C and samples were eluted at 1 ml per minute, starting with a mixture of acetonitrile containing 0.01% trifluoroacetic acid (solvent A) and 50% type I water containing 10% methanol and 0.01% trifluoroacetic acid (solvent A) for 1 min. The gradient was changed to 100% solvent A over 3 min, and further eluted for 5 min with 100% solvent A. We detected A2E and vitamin A dimers at 445 nm.

### ERG

ERG recordings were performed as previously described (Ma et al., 2011; Washington et al., 2007). For the dose-response protocol, we used flashes provided by a xenon lamp ranging from 0.0001 to 2.25 cd s/m^2^, scotopic units, in nine steps. For each animal, ERG potentials were recorded from both eyes. Traces judged excessively noisy were rejected. At least two ERG curves were averaged for each step and eye. Traces from both eyes of each animal were then averaged to give an ERG response for each probe flash for each step for each animal. Using GraphPad Prism (GraphPad Software, Inc., La Jolla, CA, USA), we then fit and averaged the a- and b-wave responses for each light intensity and for each animal to a sigmoidal curve (Low, 1987) described by: Y=Bottom+(Top-Bottom)/{1+10 ^ [(LogEC50-X)×HillSlope]}, where Top is the maximum a- or b-wave, in the same units as Y; LogEC50 is the intensity needed to achieve a half-maximum a- or b-wave, in the same units as X; HillSlope is a parameter that describes the steepness of the dose-response curve. *P* values were calculated with an *F*-Test.

For the single flash screening protocol, a single flash of 2.25 cd s/m^2^ was presented 5-10 times to each eye. Traces judged excessively noisy were rejected. For each animal, a- and b-waves for the left, A2E-treated eye were normalized to the right, control eye. We then averaged the differences between the right and left eye for each animal.

### Histology

Eyes were fixed in Excalibur's fixative (Excalibur Pathology, Inc., Norman, OK, USA), embedded in paraffin, sectioned and stained with hematoxylin and eosin using standard procedures. Eyes were sliced through the optic nerve. Four of the eight slices were then imaged 1 mm from either side of the optic nerve head at 400× and 1000× magnifications. The retinal layers were measured using ImageJ (Wayne Rasband, US National Institutes of Health, Bethesda, MD). For each eye, measurements from the four slices were averaged to give the retinal layer thickness per eye. Measurements for each eye were then averaged to give the final retinal layer thickness per experimental cohort. *P*-values were calculated with a *t*-test. The investigator was blinded to the group allocation when assessing the outcome.

### RNA profiling

Profiling of messenger RNA was done as previously described (Ma et al., 2011). We isolated total RNA from eyecups and retinas. For the control group, we isolated RNA from four biological replicates consisting of a total of ten eyes pooled into four groups (i.e. 2 eyes, 2 eyes, 3 eyes, and 3 eyes). For the A2E-treated group, a total of six eyes were used, pooled into four biological replicates (i.e. 1 eye, 1 eye, 2 eyes and 2 eyes) before isolation of total RNA.

The 167 genes screened mediating inflammation and autophagy were: Chemokine genes: *RGD1561905_predicted (C5), Ccl11, Ccl12, Ccl17, Ccl19, Ccl2, Ccl20, Ccl21, Ccl22, Ccl24, Ccl25, Ccl3, Ccl4, Ccl5, Ccl6, Ccl7, Ccl9, Cx3cl1, Cxcl1, Cxcl10, Cxcl11, Cxcl12 (Sdf1), Cxcl2, Cxcl5, Cxcl9, Il13, Pf4*. Chemokine receptors: *Ccr1, Ccr2, Ccr3, Ccr4, Ccr5, Ccr6, Ccr7, Ccr8, Ccr9, Cx3cr1, Cxcr3, Ccr10, Il8ra, Cxcr2, Xcr1*. Cytokine genes: *Ifng (IFNγ), Il10, Il11, Il13, Il15, Il16, Il17b, Il18, Il1a, Il1b, Il1f5, Il1f6, Il3, Il4, Il5, Itgam, Itgb2, Lta, Ltb, Mif, Aimp1, Spp1, Tgfb1, Tnf, Cd40lg (Tnfsf5)*. Cytokine receptors: *Ifng (IFNγ), Il10ra, Il13, Il13ra1, Il1r1, Il1r2, Il2rb, Il2rg, Il5ra, Il6r, Il6st, Tnfrsf1a (TNFR1), Tnfrsf1b (TNFR2)*. Other genes involved in the inflammatory response: *Abcf1, Bcl6, Cxcr5, C3, Casp1, Crp, Il1r1, Cxcr2, Tollip*. Genes involved in autophagic vacuole formation: *Ambra1, Atg12, Atg16l1, Atg4b, Atg4c, Atg5, Atg9a, Becn1, Ctsd, Gabarap, Gabarapl2, Irgm, Map1lc3a, Map1lc3b, Psen1, Rb1cc1, RGD1359310, Rgs19, Tm9sf1, Ulk1, Wipi1*. Genes responsible for protein targeting to membrane/vacuole: *Atg4b, Atg4c, Gabarap*. Genes responsible for protein transport: *Atg16l1, Atg16l2, Atg3, Atg4b, Atg4c, Atg7, Atg9a, Gabarap, Gabarapl2, Rab24*. Genes involved in protein ubiquitination: *Atg3, Atg7, Park2, Park7, Pink1*. Genes with protease activity: *Atg4b, Atg4c*. Co-regulators of autophagy and apoptosis: *Akt1, App, Atg12, Atg5, Bad, Bak1, Bax, Bcl2, Bcl2l1 (Bcl-x), Becn1, Bid, Bnip3, Casp3, Casp8 (Flice), Npc1, Cdkn1b (p27Kip1), Cdkn2a (p16Ink4), Cln3, Ctsb, Cxcr4, Dapk1, Eif2ak3, Fadd, Fas (Tnfrsf6), Hdac1, Htt, Ifng, Igf1, Ins2, Mapk8 (Jnk1), Mtor, Nfkb1, Park2, Pik3cg, Prkaa1, Pten, Snca, Sqstm1, Tgfb1, Tgm2, Tnf, Tnfsf10 (Trail), Tp53*. Co-regulators of autophagy and the cell cycle: *Bax, Cdkn1b (p27Kip1), Cdkn2a (p16Ink4), Mapt, Pim2, Pten, Rb1, Tgfb1, Tp53*. Autophagy induction by intracellular pathogens: *Eif2ak3, Ifng, Lamp1*. Autophagy in response to other intracellular signals: *Arsa, Ctss, Dram2 (Tmem77), Eif4g1, Esr1 (ERa), Gaa, Hdac6, Hgs, Mapk14 (p38 Mapk), Pik3c3 (Vps34), Pik3r4, Rps6kb1*. Chaperone-mediated autophagy: *Hsp90aa1*.

To determine associations and visualize networks, we used Gene Network Central [SABiosciences (QIAGEN), Venlo, The Netherlands] (http://www.sabiosciences.com/genenetwork/genenetworkcentral.php).

### Protein extraction

All procedures were performed at 4°C. Eyes were enucleated and eyecups were prepared. Each eyecup was placed into a 1.5 ml centrifuge tube containing 250 mg of 0.1 mm glass beads and 250 mg of 0.5 mm ceria stabilized zirconium oxide beads, 200 μL of ice-cold lysate buffer (1% Triton-X-100, 20 mM HEPES, 2 mM EDTA, pH 7.4) and 2 μl of 100×protease inhibitor cocktail (Cat. P8340, Sigma-Aldrich, St. Louis, MO, USA). Samples were homogenized in a bead mill homogenizer (Bullet Blender homogenizer, Next Advance, Inc.; setting speed of 8 for 10 min) then centrifuged at 20,000 ***g*** for 15 min. The supernatant containing the protein was transferred to a new tube and protein concretion was determined by measuring the absorbance at 260 and 280 nm (NanoVue Plus, GE Healthcare Bio-Sciences Corp., Piscataway, NJ, USA).

### Cytokine array

We used the Quantibody^®^ Rat Cytokine Array-3 (Cat. QAR-CYT-3-1, RayBiotech, Inc., Norcross, GA, USA) according to the manufacturer's instructions.

### 2D Gel

#### Preparation of samples

The tissue lysate buffer was exchanged into the 2D cell lysis buffer (30 mM Tris-HCl, pH 8.8, containing 7 M urea, 2 M thiourea and 4% CHAPS {3-[(3-cholamidopropyl) dimethylammonio]-1-propanesulfonate}. Protein concentration was measured using the Bio-Rad (Bio-Rad Laboratories, Inc., Hercules, CA, USA) protein assay method. The internal standard was made by mixing an equal amount of protein from each sample.

#### CyDye labeling

For each sample, 30 μg of protein was mixed with 1.0 μl of diluted CyDye (GE Healthcare Life Sciences, Piscataway, NJ, USA), and kept in the dark on ice for 30 min. Samples were labeled with Cy3 and Cy5, respectively. The labeling reaction was stopped by adding 1.0 μl of 10 mM lysine to each sample and incubating in the dark on ice for an additional 15 min. The labeled samples were then mixed together. The 2× 2D sample buffer (8 M urea, 4% CHAPS, 20 mg/ml dithiothreitol [DTT], 2% pharmalytes and a trace amount of bromophenol blue), 100 μl DeStreak rehydration solution (7 M urea, 2 M thiourea, 4% CHAPS, 20 mg/ml DTT, 1% pharmalytes and a trace amount of bromophenol blue) were added to the labeling mix to make a total volume of 250 μl for the 13 cm IPG strip. It was mixed well and spun before loading the labeled samples into the strip holder.

#### IEF and SDS-PAGE

After loading the labeled samples, isoelectric focusing (IEF) (pH 3-10 Linear) was run following the protocol provided by GE Healthcare. Upon finishing the IEF, the IPG strips were incubated in the freshly made equilibration buffer-1 (50 mM Tris-HCl, pH 8.8, containing 6 M urea, 30% glycerol, 2% SDS, a trace amount of bromophenol blue and 10 mg/ml DTT) for 15 min with gentle shaking. The strips were then rinsed in the freshly made equilibration buffer-2 (50 mM Tris-HCl, pH 8.8, containing 6 M urea, 30% glycerol, 2% SDS, a trace amount of bromophenol blue and 45 mg/ml DTT) for 10 min with gentle shaking. Next, the IPG strips were rinsed in the SDS gel running buffer before transferring into 12% SDS gels. The SDS gels were run at 15°C.

#### Image scan and data analysis

Gel images were scanned immediately following the SDS-PAGE using Typhoon TRIO (GE Healthcare). The scanned images were then analyzed by Image Quant software (version 6.0, GE Healthcare), followed by in-gel analysis using DeCyder software (version 6.5, GE Healthcare).

### Protein identification by mass spectrometry

#### Spot picking and trypsin digestion

The spots of interest were picked up by an Ettan Spot Picker (GE Healthcare Life Sciences) based on the in-gel analysis and spot picking design by DeCyder software. The gel spots were washed a few times then digested in-gel with modified porcine trypsin protease (Trypsin Gold, Promega, Madison, WI, USA). The digested tryptic peptides were desalted by ZipTip^®^ C18 (EMD Millipore, Billerica, MA, USA). Peptides were eluted from the ZipTip with 0.5 μl of matrix solution (cyano-4-hydroxycinnamic acid 5 mg/ml in 50% acetonitrile, 0.1% trifluoroacetic acid, 25 mM ammonium bicarbonate) and spotted on the AB SCIEX MALDI plate (Opti-TOF^TM^ 384 Well Insert).

#### Mass spectrometry

MALDI-TOF MS and TOF/TOF tandem MS/MS were performed on an AB SCIEX TOF/TOF™ 5800 System (AB SCIEX, Framingham, MA, USA). MALDI-TOF mass spectra were acquired in reflectron positive ion mode, averaging 4000 laser shots per spectrum. TOF/TOF tandem MS fragmentation spectra were acquired for each sample, averaging 4000 laser shots per fragmentation spectrum on each of the ten most abundant ions present in each sample (excluding trypsin autolytic peptides and other known background ions).

#### Database search

Both of the resulting peptide mass and the associated fragmentation spectra were submitted to the GPS Explorer workstation equipped Sodium iodate with the MASCOT search engine (Matrix Science, Boston, MA, USA) to search the database of the National Center for Biotechnology Information non-redundant (NCBInr). Searches were performed without constraining protein molecular weight or isoelectric point, with variable carbamidomethylation of cysteine and oxidation of methionine residues, and with one missed cleavage also allowed in the search parameters. Candidates with either protein score C.I.% or Ion C.I.% greater than 95 were considered significant.

### Statistics

Statistical analyses were performed with GraphPad Prism. Values are shown as mean±s.e.m. or s.d. A *P*-value of less than 0.05 was considered significant as calculated by a two-sided, unpaired, T- or *F*-test, where appropriate. For population proportions, we used two-tailed, Fisher's Exact Test, using 2×2 contingency tables and the method of summing small *P*-values.

## References

[BIO058600C1] Adini, I., Ghosh, K., Adini, A., Chi, Z.-L., Yoshimura, T., Benny, O., Connor, K. M., Rogers, M. S., Bazinet, L., Birsner, A. E. et al. (2014). Melanocyte-secreted fibromodulin promotes an angiogenic microenvironment. *J. Clin. Invest.* 124, 425-436. 10.1172/JCI69404 24355922 PMC3871226

[BIO058600C2] Anderson, O. A., Finkelstein, A. and Shima, D. T. (2013). A2E induces IL-1ss production in retinal pigment epithelial cells via the NLRP3 inflammasome. *PLoS ONE* 8, e67263. 10.1371/journal.pone.0067263 23840644 PMC3696103

[BIO058600C3] Avalle, L. B., Wang, Z., Dillon, J. P. and Gaillard, E. R. (2004). Observation of A2E oxidation products in human retinal lipofuscin. *Exp. Eye Res.* 78, 895-898. 10.1016/j.exer.2003.10.023 15037123

[BIO058600C4] Beattie, J. R., Pawlak, A. M., Boulton, M. E., Zhang, J., Monnier, V. M., McGarvey, J. J. and Stitt, A. W. (2010). Multiplex analysis of age-related protein and lipid modifications in human Bruch's membrane. *FASEB J.* 24, 4816-4824. 10.1096/fj.10.166090 20686107 PMC2992374

[BIO058600C5] Ben-Shabat, S., Itagaki, Y., Jockusch, S., Sparrow, J. R., Turro, N. J. and Nakanishi, K. (2002a). Formation of a nonaoxirane from A2E, a lipofuscin fluorophore related to macular degeneration, and evidence of singlet oxygen involvement. *Angew. Chem. Int. Ed. Engl.* 41, 814-817. 10.1002/1521-3773(20020301)41:5<814::AID-ANIE814>3.0.CO;2-2 12491345

[BIO058600C6] Ben-Shabat, S., Parish, C. A., Vollmer, H. R., Itagaki, Y., Fishkin, N., Nakanishi, K. and Sparrow, J. R. (2002b). Biosynthetic studies of A2E, a major fluorophore of retinal pigment epithelial lipofuscin. *J. Biol. Chem.* 277, 7183-7190. 10.1074/jbc.M108981200 11756445

[BIO058600C7] Bermann, M., Schutt, F., Holz, F. G. and Kopitz, J. (2001). Does A2E, a retinoid component of lipofuscin and inhibitor of lysosomal degradative functions, directly affect the activity of lysosomal hydrolases? *Exp. Eye Res.* 72, 191-195. 10.1006/exer.2000.0949 11161735

[BIO058600C8] Bertolotto, M., Borgia, L. and Iester, M. (2014). Hyperautofluorescence in outer retinal layers thinning. *Biomed. Res. Int.* 2014, 741538. 10.1155/2014/741538 25276816 PMC4174970

[BIO058600C9] Biesemeier, A., Taubitz, T., Julien, S., Yoeruek, E. and Schraermeyer, U. (2014). Choriocapillaris breakdown precedes retinal degeneration in age-related macular degeneration. *Neurobiol. Aging* 35, 2562-2573. 10.1016/j.neurobiolaging.2014.05.003 24925811

[BIO058600C10] Bird, A. (2020). Role of retinal pigment epithelium in age-related macular disease: a systematic review. *Br. J. Ophthalmol*. 10.1136/bjophthalmol-2020-317447 32950958

[BIO058600C11] Birnbach, C. D., Järveläínen, M., Possin, D. E. and Milam, A. H. (1994). Histopathology and immunocytochemistry of the neurosensory retina in fundus flavimaculatus. *Ophthalmology* 101, 1211-1219. 10.1016/S0161-6420(13)31725-4 8035984

[BIO058600C12] Bora, N. S., Matta, B., Lyzogubov, V. V. and Bora, P. S. (2014). Relationship between the complement system, risk factors and prediction models in age-related macular degeneration. *Mol. Immunol.* 63, 176-183. 10.1016/j.molimm.2014.07.012 25074023

[BIO058600C13] Cai, C. X., Light, J. G. and Handa, J. T. (2018). Quantifying the rate of ellipsoid zone loss in stargardt disease. *Am. J. Ophthalmol.* 186, 1-9. 10.1016/j.ajo.2017.10.032 29126757

[BIO058600C14] Camelo, S. (2014). Potential sources and roles of adaptive immunity in age-related macular degeneration: shall we rename AMD into autoimmune macular disease? *Autoimmune. Dis.* 2014, 532487. 24876950 10.1155/2014/532487 PMC4022009

[BIO058600C15] Crabb, J. W., Miyagi, M., Gu, X., Shadrach, K., West, K. A., Sakaguchi, H., Kamei, M., Hasan, A., Yan, L., Rayborn, M. E. et al. (2002). Drusen proteome analysis: an approach to the etiology of age-related macular degeneration. *Proc. Natl. Acad. Sci. USA* 99, 14682-14687. 10.1073/pnas.222551899 12391305 PMC137479

[BIO058600C16] Curcio, C. A., Zanzottera, E. C., Ach, T., Balaratnasingam, C. and Freund, K. B. (2017). Activated retinal pigment epithelium, an optical coherence tomography biomarker for progression in age-related macular degeneration. *Invest. Ophthalmol. Vis. Sci.* 58, BIO211-BIO226. 10.1167/iovs.16-19778 28785769 PMC5557213

[BIO058600C17] Dai, G., Chou, N., He, L., Gyamfi, M. A., Mendy, A. J., Slitt, A. L., Klaassen, C. D. and Wan, Y. J. (2005). Retinoid X receptor alpha Regulates the expression of glutathione s-transferase genes and modulates acetaminophen-glutathione conjugation in mouse liver. *Mol. Pharmacol.* 68, 1590-1596. 10.1124/mol.105.013680 16157696

[BIO058600C18] De, S. and Sakmar, T. P. (2002). Interaction of A2E with model membranes. Implications to the pathogenesis of age-related macular degeneration. *J. Gen. Physiol.* 120, 147-157. 10.1085/jgp.20028566 12149277 PMC2234466

[BIO058600C19] Dontsov, A. E., Sakina, N. L., Golubkov, A. M. and Ostrovsky, M. A. (2009). Light-induced release of A2E photooxidation toxic products from lipofuscin granules of human retinal pigment epithelium. *Dokl Biochem. Biophys.* 425, 98-101. 10.1134/S1607672909020112 19496332

[BIO058600C20] Eagle, R. C., Jr, Lucier, A. C., Bernardino, V. B., Jr and Yanoff, M. (1980). Retinal pigment epithelial abnormalities in fundus flavimaculatus: a light and electron microscopic study. *Ophthalmology* 87:1189-1200. 10.1016/S0161-6420(80)35106-3 6165950

[BIO058600C21] Eldred, G. E. and Katz, M. L. (1988). Fluorophores of the human retinal pigment epithelium: separation and spectral characterization. *Exp. Eye Res.* 47, 71-86. 10.1016/0014-4835(88)90025-5 3409988

[BIO058600C22] Eldred, G. E. and Lasky, M. R. (1993). Retinal age pigments generated by self-assembling lysosomotropic detergents. *Nature* 361, 724-726. 10.1038/361724a0 8441466

[BIO058600C23] Ertel, W., Kremer, J. P., Kenney, J., Steckholzer, U., Jarrar, D., Trentz, O. and Schildberg, F. W. (1995). Downregulation of proinflammatory cytokine release in whole blood from septic patients. *Blood* 85, 1341-1347. 10.1182/blood.V85.5.1341.bloodjournal8551341 7858264

[BIO058600C24] Ferris, F. L., III, Fine, S. L. and Hyman, L. (1984). Age-related macular degeneration and blindness due to neovascular maculopathy. *Arch. Ophthalmol.* 102:1640-1642. 10.1001/archopht.1984.01040031330019 6208888

[BIO058600C25] Finnemann, S. C., Leung, L. W. and Rodriguez-Boulan, E. (2002). The lipofuscin component A2E selectively inhibits phagolysosomal degradation of photoreceptor phospholipid by the retinal pigment epithelium. *Proc. Natl. Acad. Sci. USA* 99, 3842-3847. 10.1073/pnas.052025899 11904436 PMC122611

[BIO058600C26] Fishkin, N., Jang, Y.-P., Itagaki, Y., Sparrow, J. R. and Nakanishi, K. (2003). A2-rhodopsin: a new fluorophore isolated from photoreceptor outer segments. *Org. Biomol. Chem.* 1, 1101-1105. 10.1039/b212213h 12926382

[BIO058600C27] Fishkin, N. E., Sparrow, J. R., Allikmets, R. and Nakanishi, K. (2005). Isolation and characterization of a retinal pigment epithelial cell fluorophore: an all-trans-retinal dimer conjugate. *Proc. Natl. Acad. Sci. USA* 102, 7091-7096. 10.1073/pnas.0501266102 15870200 PMC1129110

[BIO058600C28] Gartner, S. and Henkind, P. (1981). Aging and degeneration of the human macula. 1. Outer nuclear layer and photoreceptors. *Br. J. Ophthalmol.* 65, 23-28. 10.1136/bjo.65.1.23 7448155 PMC1039407

[BIO058600C29] Harris, V. K., Donelan, N., Yan, Q. J., Clark, K., Touray, A., Rammal, M. and Sadiq, S. A. (2013). Cerebrospinal fluid fetuin-A is a biomarker of active multiple sclerosis. *Mult. Scler.* 19, 1462-1472. 10.1177/1352458513477923 23439582

[BIO058600C30] Hogan, M. J. (1972). Role of the retinal pigment epithelium in macular disease. *Trans. Am. Acad. Ophthalmol. Otolaryngol.* 76, 64-80. 5024602

[BIO058600C31] Holz, F. G., Schutt, F., Kopitz, J. and Volcker, H. E. (1999). [Introduction of the lipofuscin-fluorophor A2E into the lysosomal compartment of human retinal pigment epithelial cells by coupling to LDL particles. An in vitro model of retinal pigment epithelium cell aging]. *Ophthalmologe* 96, 781-785. 10.1007/s003470050496 10643311

[BIO058600C32] Iriyama, A., Fujiki, R., Inoue, Y., Takahashi, H., Tamaki, Y., Takezawa, S., Takeyama, K., Jang, W. D., Kato, S. and Yanagi, Y. (2008). A2E, a pigment of the lipofuscin of retinal pigment epithelial cells, is an endogenous ligand for retinoic acid receptor. *J. Biol. Chem.* 283, 11947-11953. 10.1074/jbc.M708989200 18326047

[BIO058600C33] Iriyama, A., Inoue, Y., Takahashi, H., Tamaki, Y., Jang, W. D. and Yanagi, Y. (2009). A2E, a component of lipofuscin, is pro-angiogenic in vivo. *J. Cell. Physiol.* 220, 469-475. 10.1002/jcp.21792 19418485

[BIO058600C34] Kaarniranta, K., Sinha, D., Blasiak, J., Kauppinen, A., Vereb, Z., Salminen, A., Boulton, M. E. and Petrovski, G. (2013). Autophagy and heterophagy dysregulation leads to retinal pigment epithelium dysfunction and development of age-related macular degeneration. *Autophagy* 9, 973-984. 10.4161/auto.24546 23590900 PMC3722332

[BIO058600C35] Kanofsky, J. R., Sima, P. D. and Richter, C. (2003). Singlet-oxygen generation from A2E. *Photochem. Photobiol.* 77, 235-242. 10.1562/0031-8655(2003)077<0235:SOGFA>2.0.CO;2 12685649

[BIO058600C36] Katz, M. L., Gao, C. L. and Rice, L. M. (1996). Formation of lipofuscin-like fluorophores by reaction of retinal with photoreceptor outer segments and liposomes. *Mech. Ageing Dev.* 92, 159-174. 10.1016/S0047-6374(96)01817-9 9080396

[BIO058600C37] Katz, M. L., Rice, L. M. and Gao, C. L. (1999). Reversible accumulation of lipofuscin-like inclusions in the retinal pigment epithelium. *Invest. Ophthalmol. Vis. Sci.* 40, 175-181. 9888441

[BIO058600C38] Kenney, M. C., Hertzog, D., Chak, G., Atilano, S. R., Khatibi, N., Soe, K., Nobe, A., Yang, E., Chwa, M., Zhu, F. et al. (2013). Mitochondrial DNA haplogroups confer differences in risk for age-related macular degeneration: a case control study. *BMC Med. Genet.* 14, 4. 10.1186/1471-2350-14-4 23302509 PMC3566905

[BIO058600C39] Lakkaraju, A., Finnemann, S. C. and Rodriguez-Boulan, E. (2007). The lipofuscin fluorophore A2E perturbs cholesterol metabolism in retinal pigment epithelial cells. *Proc. Natl. Acad. Sci. USA* 104, 11026-11031. 10.1073/pnas.0702504104 17578916 PMC1904145

[BIO058600C40] Lamin, A., Oakley, J. D., Dubis, A. M., Russakoff, D. B. and Sivaprasad, S. (2019). Changes in volume of various retinal layers over time in early and intermediate age-related macular degeneration. *Eye (Lond)* 33, 428-434. 10.1038/s41433-018-0234-9 30310161 PMC6460706

[BIO058600C41] Low, J. C. (1987). The corneal ERG of the heterozygous retinal degeneration mouse. *Graefes Arch. Clin. Exp. Ophthalmol.* 225, 413-417. 10.1007/BF02334167 2824296

[BIO058600C42] Lukiw, W. J., Mukherjee, P. K., Cui, J. G. and Bazan, N. G. (2006). A2E selectively induces cox-2 in ARPE-19 and human neural cells. *Curr. Eye Res.* 31, 259-263. 10.1080/02713680600556974 16531283

[BIO058600C43] Ma, L., Kaufman, Y., Zhang, J. and Washington, I. (2011). C20-D3-vitamin A slows lipofuscin accumulation and electrophysiological retinal degeneration in a mouse model of Stargardt disease. *J. Biol. Chem.* 286, 7966-7974. 10.1074/jbc.M110.178657 21156790 PMC3048683

[BIO058600C44] Ma, W., Coon, S., Zhao, L., Fariss, R. N. and Wong, W. T. (2013). A2E accumulation influences retinal microglial activation and complement regulation. *Neurobiol. Aging* 34, 943-960. 10.1016/j.neurobiolaging.2012.06.010 22819137 PMC3480997

[BIO058600C45] Machalinska, A., Lubinski, W., Klos, P., Kawa, M., Baumert, B., Penkala, K., Grzegrzolka, R., Karczewicz, D., Wiszniewska, B. and Machalinski, B. (2010). Sodium iodate selectively injuries the posterior pole of the retina in a dose-dependent manner: morphological and electrophysiological study. *Neurochem. Res.* 35, 1819-1827. 10.1007/s11064-010-0248-6 20725778 PMC2957578

[BIO058600C46] Maeda, H., Ogata, N., Yi, X., Takeuchi, M., Ohkuma, H. and Uyama, M. (1998). Apoptosis of photoreceptor cells in ornithine-induced retinopathy. *Graefes Arch. Clin. Exp. Ophthalmol.* 236, 207-212. 10.1007/s004170050066 9541825

[BIO058600C47] Mihai, D. M. and Washington, I. (2014). Vitamin A dimers trigger the protracted death of retinal pigment epithelium cells. *Cell Death Dis.* 5, e1348. 10.1038/cddis.2014.314 25058422 PMC4123103

[BIO058600C48] Moiseyev, G., Nikolaeva, O., Chen, Y., Farjo, K., Takahashi, Y. and Ma, J. X. (2010). Inhibition of the visual cycle by A2E through direct interaction with RPE65 and implications in Stargardt disease. *Proc. Natl. Acad. Sci. USA* 107, 17551-17556. 10.1073/pnas.1008769107 20876139 PMC2955102

[BIO058600C49] Murdaugh, L. S., Dillon, J. and Gaillard, E. R. (2009). Modifications to the basement membrane protein laminin using glycolaldehyde and A2E: a model for aging in Bruch's membrane. *Exp. Eye Res.* 89, 187-192. 10.1016/j.exer.2009.03.021 19358843

[BIO058600C50] Murdaugh, L. S., Wang, Z., Del Priore, L. V., Dillon, J. and Gaillard, E. R. (2010a). Age-related accumulation of 3-nitrotyrosine and nitro-A2E in human Bruch's membrane. *Exp. Eye Res.* 90, 564-571. 10.1016/j.exer.2010.01.014 20153746

[BIO058600C51] Murdaugh, L. S., Avalle, L. B., Mandal, S., Dill, A. E., Dillon, J., Simon, J. D. and Gaillard, E. R. (2010b). Compositional studies of human RPE lipofuscin. *J. Mass Spectrom.* 45, 1139-1147. 10.1002/jms.1795 20860013

[BIO058600C52] Murdaugh, L. S., Mandal, S., Dill, A. E., Dillon, J., Simon, J. D. and Gaillard, E. R. (2011). Compositional studies of human RPE lipofuscin: mechanisms of molecular modifications. *J. Mass Spectrom.* 46, 90-95. 10.1002/jms.1865 21182214

[BIO058600C53] Nordgaard, C. L., Berg, K. M., Kapphahn, R. J., Reilly, C., Feng, X., Olsen, T. W. and Ferrington, D. A. (2006). Proteomics of the retinal pigment epithelium reveals altered protein expression at progressive stages of age-related macular degeneration. *Invest. Ophthalmol. Vis. Sci.* 47, 815-822. 10.1167/iovs.05-0976 16505012

[BIO058600C54] Nowak, J. Z. (2013). Oxidative stress, polyunsaturated fatty acids-derived oxidation products and bisretinoids as potential inducers of CNS diseases: focus on age-related macular degeneration. *Pharmacol. Rep.* 65, 288-304. 10.1016/S1734-1140(13)71005-3 23744414

[BIO058600C55] Penn, J., Mihai, D. M. and Washington, I. (2014). Morphologic and physiologic retinal degeneration induced by intravenous delivery of vitamin A dimers in the leporid retina. *Dis. Model. Mech.* 8, 131-138. 25504631 10.1242/dmm.017194 PMC4314778

[BIO058600C56] Penn, J., Mihai, D. M. and Washington, I. (2015). Morphological and physiological retinal degeneration induced by intravenous delivery of vitamin A dimers in rabbits. *Dis. Model. Mech.* 8, 131-138. 10.1242/dmm.017194 25504631 PMC4314778

[BIO058600C57] Radu, R. A., Mata, N. L., Bagla, A. and Travis, G. H. (2004). Light exposure stimulates formation of A2E oxiranes in a mouse model of Stargardt's macular degeneration. *Proc. Natl. Acad. Sci. USA* 101, 5928-5933. 10.1073/pnas.0308302101 15067110 PMC395900

[BIO058600C58] Reinboth, J. J., Gautschi, K., Munz, K., Eldred, G. E. and Reme, C. E. (1997). Lipofuscin in the retina: quantitative assay for an unprecedented autofluorescent compound (pyridinium bis-retinoid, A2-E) of ocular age pigment. *Exp. Eye Res.* 65, 639-643. 10.1006/exer.1997.0367 9367643

[BIO058600C59] Roberts, J. E., Kukielczak, B. M., Hu, D. N., Miller, D. S., Bilski, P., Sik, R. H., Motten, A. G. and Chignell, C. F. (2002). The role of A2E in prevention or enhancement of light damage in human retinal pigment epithelial cells. *Photochem. Photobiol.* 75, 184-190. 10.1562/0031-8655(2002)075<0184:TROAIP>2.0.CO;2 11883606

[BIO058600C60] Ryals, R. C., Andrews, M. D., Datta, S., Coyner, A. S., Fischer, C. M., Wen, Y., Pennesi, M. E. and McGill, T. J. (2017). Long-term characterization of retinal degeneration in royal college of surgeons rats using spectral-domain optical coherence Tomography. *Invest. Ophthalmol. Vis. Sci.* 58, 1378-1386. 10.1167/iovs.16-20363 28253400 PMC5361458

[BIO058600C61] Sakai, N., Decatur, J., Nakanishi, K. and Eldred, G. E. (1996). Ocular age pigment ‘'A2-E'': an unprecedented pyridinium bisretinoid. *J. Am. Chem. Soc.* 118, 1559-1560. 10.1021/ja953480g

[BIO058600C62] Sarks, S. H. (1976). Ageing and degeneration in the macular region: a clinico-pathological study. *Br. J. Ophthalmol.* 60, 324-341. 10.1136/bjo.60.5.324 952802 PMC1042725

[BIO058600C63] Sarks, J. P., Sarks, S. H. and Killingsworth, M. C. (1988). Evolution of geographic atrophy of the retinal pigment epithelium. *Eye (Lond)* 2:552-577. 10.1038/eye.1988.106 2476333

[BIO058600C64] Sokolov, V. S., Sokolenko, E. A., Sokolov, A. V., Dontsov, A. E., Chizmadzhev, Y. A. and Ostrovsky, M. A. (2007). Interaction of pyridinium bis-retinoid (A2E) with bilayer lipid membranes. *J. Photochem. Photobiol. B* 86, 177-185. 10.1016/j.jphotobiol.2006.09.006 17070694

[BIO058600C65] Sparrow, J. R. (2010). Bisretinoids of RPE lipofuscin: trigger for complement activation in age-related macular degeneration. *Adv. Exp. Med. Biol.* 703, 63-74. 10.1007/978-1-4419-5635-4_5 20711707

[BIO058600C66] Sparrow, J. R. and Cai, B. (2001). Blue light-induced apoptosis of A2E-containing RPE: involvement of caspase-3 and protection by Bcl-2. *Invest. Ophthalmol. Vis. Sci.* 42, 1356-1362. 11328751

[BIO058600C67] Sparrow, J. R., Parish, C. A., Hashimoto, M. and Nakanishi, K. (1999). A2E, a lipofuscin fluorophore, in human retinal pigmented epithelial cells in culture. *Invest. Ophthalmol. Vis. Sci.* 40, 2988-2995. 10549662

[BIO058600C68] Sparrow, J. R., Nakanishi, K. and Parish, C. A. (2000). The lipofuscin fluorophore A2E mediates blue light-induced damage to retinal pigmented epithelial cells. *Invest. Ophthalmol. Vis. Sci.* 41, 1981-1989. 10845625

[BIO058600C69] Sparrow, J. R., Zhou, J. and Cai, B. (2003a). DNA is a target of the photodynamic effects elicited in A2E-laden RPE by blue-light illumination. *Invest. Ophthalmol. Vis. Sci.* 44, 2245-2251. 10.1167/iovs.02-0746 12714667

[BIO058600C70] Sparrow, J. R., Vollmer-Snarr, H. R., Zhou, J., Jang, Y. P., Jockusch, S., Itagaki, Y. and Nakanishi, K. (2003b). A2E-epoxides damage DNA in retinal pigment epithelial cells. Vitamin E and other antioxidants inhibit A2E-epoxide formation. *J. Biol. Chem.* 278, 18207-18213. 10.1074/jbc.M300457200 12646558

[BIO058600C71] Sparrow, J. R., Zhou, J., Ben-Shabat, S., Vollmer, H., Itagaki, Y. and Nakanishi, K. (2002). Involvement of oxidative mechanisms in blue-light-induced damage to A2E-laden RPE. *Invest. Ophthalmol. Vis. Sci.* 43, 1222-1227. 11923269

[BIO058600C72] Sparrow, J. R., Cai, B., Jang, Y. P., Zhou, J. and Nakanishi, K. (2006). A2E, a fluorophore of RPE lipofuscin, can destabilize membrane. *Adv. Exp. Med. Biol.* 572, 63-68. 10.1007/0-387-32442-9_10 17249556

[BIO058600C73] Tabatabay, C. A., D'Amico, D. J., Hanninen, L. A., Casey, V. N. and Kenyon, K. R. (1987). Residual bodies in the retinal pigment epithelium induced by intravitreal netilmicin. *Invest. Ophthalmol. Vis. Sci.* 28, 1783-1787. 2822595

[BIO058600C74] Tezel, T. H., Geng, L., Lato, E. B., Schaal, S., Liu, Y., Dean, D., Klein, J. B. and Kaplan, H. J. (2009). Synthesis and secretion of hemoglobin by retinal pigment epithelium. *Invest. Ophthalmol. Vis. Sci.* 50, 1911-1919. 10.1167/iovs.07-1372 19060278

[BIO058600C75] Thao, M. T., Renfus, D. J., Dillon, J. and Gaillard, E. R. (2013). A2E mediated photochemical modification to fibronectin and its implications to age related changes in Bruch's membrane. *Photochem. Photobiol.* 90, 329-334. 10.1111/php.12200 24303925

[BIO058600C76] Toprak, I., Yaylali, V. and Yildirim, C. (2017). Early deterioration in ellipsoid zone in eyes with non-neovascular age-related macular degeneration. *Int. Ophthalmol.* 37, 801-806. 10.1007/s10792-016-0331-3 27591785

[BIO058600C77] Ueda, K., Zhao, J., Kim, H. J. and Sparrow, J. R. (2016). Photodegradation of retinal bisretinoids in mouse models and implications for macular degeneration. *Proc. Natl. Acad. Sci. USA* 113, 6904-6909. 10.1073/pnas.1524774113 27274068 PMC4922174

[BIO058600C78] Ugurlu, N., Asik, M. D., Yulek, F., Neselioglu, S. and Cagil, N. (2013). Oxidative stress and anti-oxidative defence in patients with age-related macular degeneration. *Curr. Eye Res.* 38, 497-502. 10.3109/02713683.2013.774023 23432778

[BIO058600C79] van der Burght, B. W., Hansen, M., Olsen, J., Zhou, J., Wu, Y., Nissen, M. H. and Sparrow, J. R. (2013). Early changes in gene expression induced by blue light irradiation of A2E-laden retinal pigment epithelial cells. *Acta Ophthalmol.* 91, e537-e545. 10.1111/aos.12146 23742627 PMC4955808

[BIO058600C80] Vives-Bauza, C., Anand, M., Shiraz, A. K., Magrane, J., Gao, J., Vollmer-Snarr, H. R., Manfredi, G. and Finnemann, S. C. (2008). The age lipid A2E and mitochondrial dysfunction synergistically impair phagocytosis by retinal pigment epithelial cells. *J. Biol. Chem.* 283, 24770-24780. 10.1074/jbc.M800706200 18621729 PMC2529005

[BIO058600C81] Wang, Z., Keller, L. M., Dillon, J. and Gaillard, E. R. (2006). Oxidation of A2E results in the formation of highly reactive aldehydes and ketones. *Photochem. Photobiol.* 82, 1251-1257. 10.1562/2006-04-01-RA-864 16813456

[BIO058600C82] Washington, I., Jockusch, S., Itagaki, Y., Turro, N. J. and Nakanishi, K. (2005). Superoxidation of bisretinoids. *Angew. Chem. Int. Ed. Engl.* 44, 7097-7100. 10.1002/anie.200501346 16222651

[BIO058600C83] Washington, I., Turro, N. J. and Nakanishi, K. (2006). Superoxidation of retinoic acid. *Photochem. Photobiol.* 82, 1394-1397. 10.1111/j.1751-1097.2006.tb09790.x 16608387

[BIO058600C84] Washington, I., Zhou, J., Jockusch, S., Turro, N. J., Nakanishi, K. and Sparrow, J. R. (2007). Chlorophyll derivatives as visual pigments for super vision in the red. *Photochem. Photobiol. Sci.* 6, 775-779. 10.1039/b618104j 17609771

[BIO058600C85] Whitmore, S. S., Fortenbach, C. R., Cheng, J. L., DeLuca, A. P., Critser, D. B., Geary, E. L., Hoffmann, J. M., Stone, E. M. and Han, I. C. (2020). Analysis of retinal sublayer thicknesses and rates of change in ABCA4-associated Stargardt disease. *Sci. Rep.* 10, 16576. 10.1038/s41598-020-73645-5 33024232 PMC7538899

[BIO058600C86] Wielgus, A. R., Collier, R. J., Martin, E., Lih, F. B., Tomer, K. B., Chignell, C. F. and Roberts, J. E. (2010). Blue light induced A2E oxidation in rat eyes--experimental animal model of dry AMD. *Photochem. Photobiol. Sci.* 9, 1505-1512. 10.1039/c0pp00133c 20922251 PMC12384401

[BIO058600C87] Wu, Y., Yanase, E., Feng, X., Siegel, M. M. and Sparrow, J. R. (2010). Structural characterization of bisretinoid A2E photocleavage products and implications for age-related macular degeneration. *Proc. Natl. Acad. Sci. USA* 107, 7275-7280. 10.1073/pnas.0913112107 20368460 PMC2867734

[BIO058600C88] Yasukawa, T., Wiedemann, P., Hoffmann, S., Kacza, J., Eichler, W., Wang, Y. S., Nishiwaki, A., Seeger, J. and Ogura, Y. (2007). Glycoxidized particles mimic lipofuscin accumulation in aging eyes: a new age-related macular degeneration model in rabbits. *Graefes Arch. Clin. Exp. Ophthalmol.* 245, 1475-1485. 10.1007/s00417-007-0571-z 17406884

[BIO058600C89] Yoon, K. D., Yamamoto, K., Zhou, J. and Sparrow, J. R. (2011). Photo-products of retinal pigment epithelial bisretinoids react with cellular thiols. *Mol. Vis.* 17, 1839-1849. 21850158 PMC3137558

[BIO058600C90] Yoon, K. D., Yamamoto, K., Ueda, K., Zhou, J. and Sparrow, J. R. (2012). A novel source of methylglyoxal and glyoxal in retina: implications for age-related macular degeneration. *PLoS ONE* 7, e41309. 10.1371/journal.pone.0041309 22829938 PMC3400616

[BIO058600C92] Zhang, D., Robinson, K., Saad, L. and Washington, I. (2021). Vitamin A cycle byproducts impede dark adaptation. *J. Biol. Chem*. 297, 101074. 10.1016/j.jbc.2021.101074 34391781 PMC8427233

[BIO058600C91] Zhou, J., Jang, Y. P., Kim, S. R. and Sparrow, J. R. (2006). Complement activation by photooxidation products of A2E, a lipofuscin constituent of the retinal pigment epithelium. *Proc. Natl. Acad. Sci. USA* 103, 16182-16187. 10.1073/pnas.0604255103 17060630 PMC1637557

